# Effects of frailty on postoperative clinical outcomes of aneurysmal subarachnoid hemorrhage: results from the National Inpatient Sample database

**DOI:** 10.1186/s12877-022-03141-0

**Published:** 2022-05-28

**Authors:** Yubin Guo, Hui Wu, Wenhua Sun, Xiang Hu, Jiong Dai

**Affiliations:** 1 Department of Neurosurgery, Renji Hospital, Shanghai Jiaotong University School of Medicine, No.1630 Dongfang Road, Shanghai, 200127 China; 2 Cerebrovascular Disease Center, Renji Hospital, Shanghai Jiaotong University School of Medicine, No.2000 Jiangyue Road, Shanghai, 201112 China

**Keywords:** Frailty, Aneurysmal subarachnoid hemorrhage (aSAH), Postoperative outcomes, National inpatient sample (NIS)

## Abstract

**Background:**

This study aimed to investigate the potential effect of preoperative frailty on postoperative clinical outcomes of patients with aneurysmal subarachnoid hemorrhage (aSAH).

**Methods:**

Data of patients aged 18 years and older who were diagnosed with subarachnoid hemorrhage or intracerebral hemorrhage, underwent aneurysm repair surgical intervention from 2005 to 2014. A retrospective database analysis was performed based on U.S. National Inpatient Sample (NIS) from 2005 to 2014. Frailty was determined using the Johns Hopkins Adjusted Clinical Groups (ACG) frailty-defining diagnoses indicator. Patients were stratified into frail and non-frail groups and the study endpoints were incidence of postoperative complications and related adverse clinical outcomes.

**Results:**

Among 20,527 included aSAH patients, 2303 (11.2%) were frail and 18,224 (88.8%) were non-frail. Significant differences were found between frailty and non-frailty groups in the four clinical outcomes (all *p* < 0.05). Multivariate analysis showed that frailty was associated with significant higher risks of discharge to institutional care (aOR: 2.50, 95%CI: 2.10–2.97), tracheostomy or gastrostomy tube replacement (aOR: 4.41, 95%CI: 3.81–5.10) and postoperative complications (aOR: 3.29, 95%CI: 2.55–4.25) but a lower risk of death in hospital (aOR: 0.40, 95%CI: 0.33–0.49) as compared with non-frailty. Stratified analysis showed the impact of frailty on some of the outcomes were greater among patients younger than 65 years than their older counterparts.

**Conclusions:**

Frailty is significantly correlated with the increased risk of discharge to institutional care, tracheostomy or gastrostomy tube placement, and postoperative complications but with the reduced risk of in-hospital mortality outcomes after aneurysm repair. Frailty seems to have greater impact among younger adults than older ones. Baseline frailty evaluation could be applied to risk stratification for aSAH patients who were undergoing surgery.

## Background

Frailty is increasingly recognized as an independent determinant of health status in older adults and, though distinct from comorbidities and disabilities, often shares ground with these factors and is associated with high morbidity and mortality in advanced age [1]. Studies have shown that frailty has a more significant predictive role in determining unfavorable outcomes than advanced age itself [2, 3]. Frailty, usually understood as a senile syndrome of decreased physiological reserve and multi-system dysregulation, is associated with increased vulnerability to stressors [4]. It is found in 2 out of every 10 adults aged 65 years and older and associated with adverse outcomes such as limited physical activity, falls, emergency department visits, greater likelihood of institutional care, and poor quality of life among community-dwelling older peoples [5]. Frailty is considered a valid predictor of adverse postoperative clinical outcomes and linked with perioperative morbidity and mortality in various surgical settings, including emergent or non-emergent general surgery [6], major and minimally invasive cardiac surgery [7, 8], cranial neurosurgery [9, 10], and transsphenoidal pituitary surgery [11]. Frailty is also noted for risk prediction in critically ill patients with various diagnoses, including stroke and intensive care patients requiring organ support [12, 13]. With respect to stroke, a previous single center study has documented that frailty was present in about one in four acute stroke patients, while a frailty syndrome was seen in three out of four patients when including pre-frailty [14]. Moreover, pre-stroke frailty was linked to severity of acute stroke in the elderly, and still more studies are needed to clarify the association between frailty and stroke prognosis [15].

Aneurysmal subarachnoid hemorrhage (aSAH) is a devastating condition with a high risk of mortality and morbidity among those who survive the first hemorrhage. It is reported that the fatality rate of aSAH patients around the world is between 27 and 44%, which represents a decreased trend since the 1990s (range, 8.3–66.7%), despite the influence of population aging on aSAH outcomes [16]. Although aggressive management is credited with reducing mortality rates [16, 17], the morbidity rate remains higher among survivors, including neurologic deficits that significantly limit subsequent physical and mental health status [18]. Only a few patients who endured aSAH resumed their routine daily activities before hemorrhage [16–18]. Multiple prognostic factors, both pre-and post-admission, are shown to contribute to unfavorable outcomes associated with aSAH, including advanced age, clinical status on admission, the severity of bleed, arterial hypertension, size, and location of the aneurysm, comorbidities, and secondary complications [18–20]. Most prognostic factors present on admission are modifiable, but those occurring during admission contribute to outcomes and are shown to be more easily influenced by treatment [20].

Nevertheless, although multiple shreds of evidence from prior studies have shown that frailty is an independent predictor of unfavorable surgical outcomes, or during critical illness [2, 3, 6–8], few studies have addressed the impact of frailty on aSAH outcomes. Thus, identifying frailty in aSAH patients may help control the increased risk of adverse outcomes and support care planning and management. Therefore, we aimed to identify the relationship between frailty on adverse outcomes of patients with aSAH in this study.

## Methods

### Study design and data source

This study extracted all data from the National Inpatient Sample (NIS) database, the largest nationwide all-payer and continuous inpatient care database in the United States (US). The NIS is sampled from individual statewide inpatient databases, including annual inpatient data from about 1050 participating hospitals in 44 states of the US, representing a 20% stratified sample of US community hospitals as determined by the American Hospital Association (AHA). It includes approximately 8 million hospitalizations each year [21], and after weighted national estimates to identify weighing, it covers more than 35 million hospitalizations nationwide. The database is administered by the Healthcare Cost and Utilization Project (HCUP) of the US National Institutes of Health (NIH). Patient data contain demographics, primary and secondary diagnoses and procedures, admission and discharge status, expected payment source, hospital characteristics, and duration of hospital stay.

### Ethics statement

All data were acquired from the Healthcare Cost and Utilization Project (HCUP) Central Distributor (https://www.distributor.hcup-us.ahrq.gov/; the certificate no. HCUP-833FWV78H). This study conforms to the NIS data-use agreement from the HCUP. The study protocol was reviewed by the Institutional Review Board (IRB) of the Renji Hospital, Shanghai Jiaotong University School of Medicine, which exempted the study from IRB approval. Since all the data in the NIS database are de-identified, the requirement for obtaining informed consent was also waived. All methods were performed in accordance with the relevant guidelines and regulations.

### Study population

**Fig. 1:**
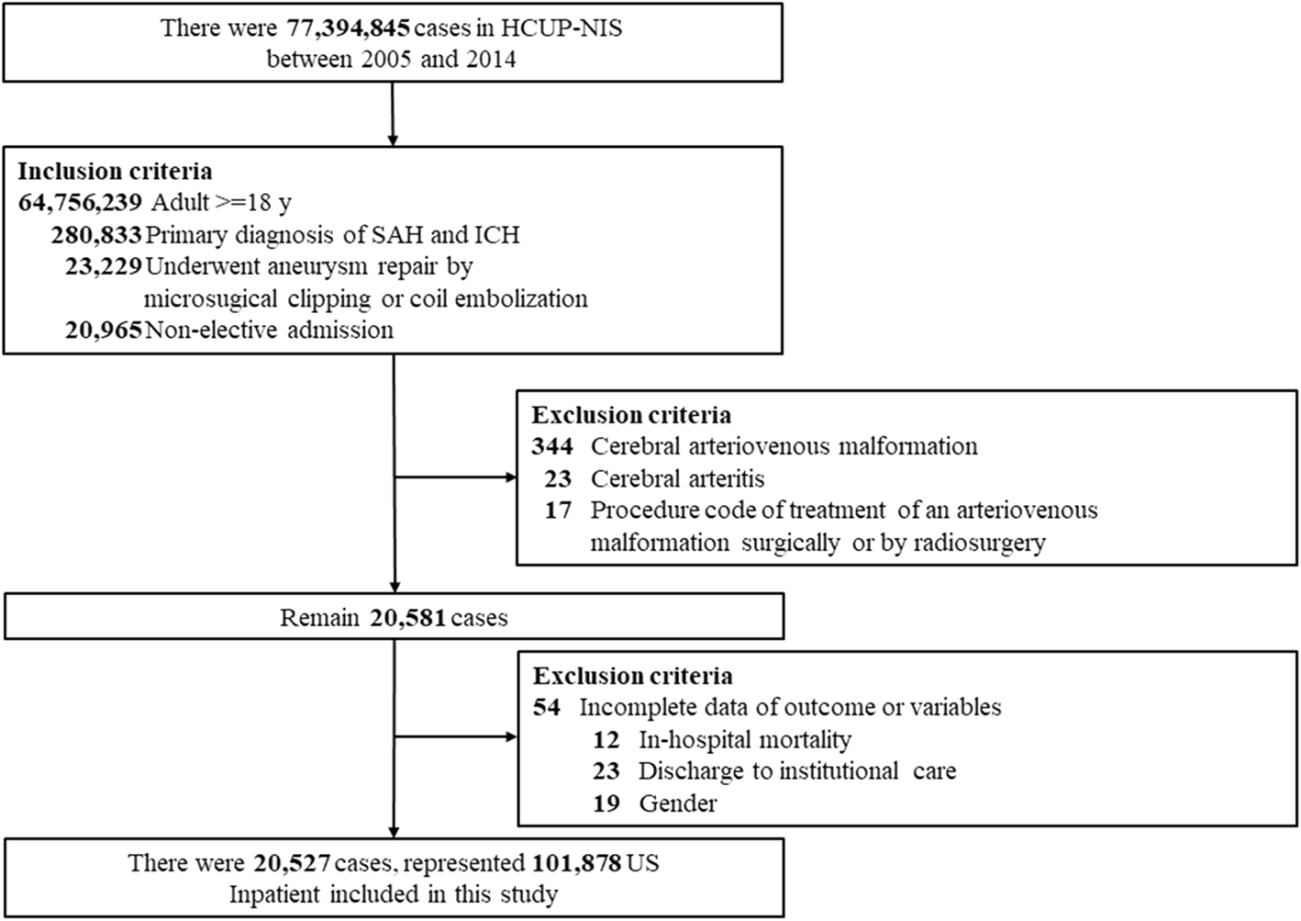
Flowchart of study subject selection. aSAH, aneurysmal subarachnoid hemorrhage; SICH, spontaneous intracerebral hemorrhage; HCUP, Healthcare Cost and Utilization Project; NIS, Nationwide Inpatient Sample

Adults aged 18 or older (≥18 years) were admitted to US hospitals from 2005 to 2014 with a primary diagnosis of subarachnoid hemorrhage according to the International Classification of Disease, Ninth Revision (ICD-9, CM code: 430) and intracerebral hemorrhage (ICD-9-CM code: 431, 432.9), who underwent aneurysm repair by microsurgical clipping or coil embolization (ICD-9 procedure code 39.51, 39.72, 39.75, 39.76, 39.79) were discovered from the NIS database. Participants only with non-elective (emergency) hospital admission were included. Patients with cerebral arteriovenous malformation (AVM) (ICD-9-CM code: 747.81, ICD-9 procedure code: 39.53), cerebral arteritis (437.4), and those who received radiosurgery (ICD-9 procedure code: 923.x) were excluded. The patients who underwent both coiling and microsurgical clipping were also excluded. Besides, we also excluded patients without complete data for primary outcomes and variables of interest in this cohort. Figure 1 shows the selection process of included patients in this study.

The participants were divided into frail and non-frail groups based on the Johns Hopkins Adjusted Clinical Groups frailty indicator [22]. A binary variable with 10 clusters of diagnoses was assigned using ICD-9 codes during admission; an instrument validated for research using administrative data [2, 23]. ACG frailty indicators include malnutrition, dementia, severely impaired vision, decubitus ulcer, urinary and fecal incontinence, weight loss, poverty, barriers to access care, and walking difficulties. Having any one of the indicators was allocated to the frailty group. The details of relevant ACG codes for frailty are shown in Table 1.

**Table 1:** ICD-9 codes for defining frailty

Variable	Diagnoses	ICD9
Malnutrition	Nutritional marasmus Other severe protein-calorie malnutrition	261, 262, 263.8, 263.9, V77.2,
Dementia	Senile dementia with delusional or depressive features Senile dementia with delirium	290.20, 290.21, 290.3
Severe vision impairment	Profound impairment, both eyes Moderate or severe impairment, better eye/lesser eye: profound	369.0, 369.00, 369.01, 369.03, 369.04, 369.06, 369.07, 369.08,
Decubitus ulcer	Decubitus ulcer	707.0, 707.00, 707.01, 707.02, 707.03, 707.04, 707.05, 707.06, 707.07, 707.09, 707.20, 707.21, 707.22, 707.23, 707.24, 707.25
Loss of weight	Abnormal loss of weight and underweight Feeding difficulties and mismanagement	783.2, 783.21, 783.22, 783.3
Incontinence	Incontinence of feces	787.6
	Urinary incontinence Continuous leakage	788.34, 788.37
Poverty	Lack of housing Inadequate housing Inadequate material resources	V60.0, V60.1 V60.2
Barriers to Access of Care	No Med Facility For Care No Med Facilities Necessary	V63.1, V63.8, V63.9
Difficulty in walking	Difficulty in walking Abnormality of gait	719.7, 781.2
Falling	Falling on stairs or steps Falling from wheelchair	E880, E880.0, E880.1, E880.9, E884.3

Data source: Abrams C, Lieberman R, Weiner JP. Development and Evaluation of The Johns Hopkins University Risk Adjustment Models for Medicare+Choice Plan Payment. Johns Hopkins University; 2003

### Study variables

#### Dependent variables

The primary endpoint was the incidence of adverse postoperative outcomes, including:

1. In-hospital mortality rate
2. Discharge to institutional care (nursing facility, extended care service facility, or hospice);
3. Tracheostomy or gastrostomy tube placement: as defined by ICD-9-CM procedures codes: 31.1, 31.2. 31.21, 31.29, 43.1, 43.11, 43.19, 44.32, 44.38, 44.39;
4. Postoperative complications: as defined by ICD-9-CM codes, including neurological (seizures 345.xx, stroke 433.x or 434.x, transient cerebral ischemia (TIA), 435.x, neurological complications after surgical procedure 997.01 or 997.09), cardiac (248.xx, 410.xx, 427.5, or 785.xx), pulmonary (512.x, 514.x, or 518.xx), renal (584.x), gastrointestinal (5601, 578.x, or 00845), venous thromboembolic (415.x or 453.x), hematological (red blood cell transfusion 94.04, 285.x, or 998.1x), sodium disturbance (253.5, 253.6, 276.0, or 276.1), and infection (041.x, 38.x, 320.x, 324.1, 481–486, 507.0, 595.0, 790.7, 995.9x, 996.64, 997.31, 998.59, or 999.31).

#### Covariates

##### Independent variables

*Demography:* Demographic variables included: age (18–45, 46–64, ≥65), gender (male, female), race/ethnicity (categorized as White, Black, Hispanic, Asian or Pacific Islander, Others based on NIS data), household income (quartile classification of estimated median household income of the patient’s ZIP Code with quartiles identified by values of 1 to 4, indicating the lowest to highest income).

*Diseases/comorbidities*: Treatment methods of aneurysm are divided into two types (Coiling or Microsurgical clipping), cerebral herniation (Dxn = 348.4), cerebral edema (Dxn = 348.5), the severity of *aSAH* (detailed description in the next paragraph), and associated comorbidities, such as Diabetes (CM_DM, CM_DMCX), Hypertension (CM_HTN_C), and Peripheral vascular disorders (CM_PERIVASC), which were obtained according to the comorbidities measures of Agency for Healthcare Research and Quality (AHRQ). Complete documentation on the Severity Measures or comorbidity measures is available on the NIS/HCUP Database Documentation websites.

*aSAH severity:* The Nationwide Inpatient Sample Subarachnoid Severity Scale (NIS-SSS) developed by Washington et al. [24] was used to determine aSAH severity. The NIS-SSS was independently validated to be equivalent to the classification of aSAH by the Hunt & Hess scale [25] for predicting six-month functional outcomes based on the Modified Rankin Score. Variables included in the NIS-SSS are coma/ stupor (ICD-9-CM 780.01, 780.03, 780.02, 780.09), hydrocephalus (ICD-9-CM 331.3, 331.4), Paresis/ plegia (438.2–438.53, 781.4), aphasia (438.1–438.89), cranial nerve deficits (378.5–378.56, 379.4–379.43), mechanical ventilation (ICD-9 procedure code: 96.04, 96.7–96.72), and Ventriculostomy/CSF shunting (ICD-9 procedure code: 02.2, 02.31–02.39). A higher severity score is indicating greater severity.

*Providers:* Provider characteristics, such as hospital size was based on the number of beds using NIS criteria size (categorized as small, medium, or large), location (rural vs. urban), and teaching status of the hospital was obtained from NIS Hospital File Data.

### Statistical analysis

Continuous data were presented as the mean ± standard error (SE) and were compared using the PROC SURVEYREG statement to test the difference between groups. Categorical data were presented as unweighted counts (weighted percentage) and were compared using the PROC SURVEYFREQ statement. The univariate and multivariate analyses were performed to determine associations among frailty and incidence of in-hospital mortality rate, discharge to institutional care, tracheostomy or gastrostomy tube placement, and any postoperative complications using the PROC SURVEYLOGISTIC statement and were presented as odds ratios (ORs) and 95% confidence intervals (CIs). Further stratified analyses were performed based on age and types of aneurysm repair surgeries performed. Since the NIS database covers 20% samples of the USA annual inpatient admissions, weighted samples (DISCWT), stratum (NIS_STRATUM), cluster (HOSPID) were used to generate national estimates for all analyses. All statistical analyses were performed using SAS 9.4 software (SAS, Cary, NC, USA). Two-sided *p*-values less than 0.05 (*p* < 0.05) were considered statistically significant.

## Results

The data of 280,833 hospitalized patients aged 18 years or older diagnosed primarily with SAH were initially extracted from the HCUP-NIS (2005–2014) database. Of these, 20,965 patients admitted emergently had undergone aneurysm repair either by microsurgical clipping or coil embolization procedure during hospitalization for medical illness. After excluding patients with the following exclusion criteria: cerebral arteriovenous malformation (*n* = 344), cerebral arteritis (*n* = 23), or radiosurgery (*n* = 17), or those without complete data for primary outcomes and variables of interest (*n* = 54), data of the remaining 20,527 patients (representing 101,878 US inpatients) formed the analytic sample (Fig. 1).

Inpatient outcomes are summarized in Table 2. Of these, 2633 (12.8%), 7843 (44.0%), 3569 (17.4%), and 16,348 (79.7%) of patients died in-hospital mortality, discharged to institutional care, underwent tracheostomy or gastrostomy tube placement, and had at least one postoperative complication, respectively. Again, statically significant differences were found between the frailty and non-frailty groups, including 10.2% vs. 13.1% with in-hospital mortality, 72.8% vs. 40.2% with discharge to institutional care, 46.8% vs. 13.7% with tracheostomy or gastrostomy tube placement, and 95.3% vs. 77.7% with at least one postoperative complication (all *P* < 0.001).

**Table 2:** Frequency of adverse outcomes of hospitalized patients with aSAH

Study outcomes	Total	Frailty	Non-frailty	***P***-value
	(***n*** = 20,527)	(***n*** = 2303)	(***n*** = 18,224)	
**Outcomes**				
In-hospital mortality	2633 (12.8)	236 (10.2)	2397 (13.1)	**< 0.001**
Discharge to institutional care	7843 (44.0)	1503 (72.8)	6340 (40.2)	**< 0.001**
Tracheostomy or gastrostomy tube placement	3569 (17.4)	1080 (46.8)	2489 (13.7)	**< 0.001**
Postoperative complications	16,348 (79.7)	2194 (95.3)	14,154 (77.7)	**< 0.001**
Neurological	6033 (29.4)	822 (35.7)	5211 (28.6)	**< 0.001**
Cardiac	1856 (9.1)	328 (14.3)	1528 (8.4)	**< 0.001**
Pulmonary	9406 (45.9)	1697 (73.6)	7709 (42.3)	**< 0.001**
Renal	1106 (5.4)	246 (10.8)	860 (4.7)	**< 0.001**
Gastrointestinal	1156 (5.6)	299 (13.0)	857 (4.7)	**< 0.001**
Venous thromboembolic	1866 (9.2)	383 (16.8)	1483 (8.2)	**< 0.001**
Hematological	4894 (23.9)	921 (40.1)	3973 (21.9)	**< 0.001**
Sodium disturbance	7325 (35.7)	1097 (47.6)	6228 (34.1)	**< 0.001**
Infectious	7708 (37.6)	1415 (61.3)	6293 (34.6)	**< 0.001**

Categorical data were presented as unweighted counts (weighted percentage)

*aSAH* aneurysmal subarachnoid hemorrhage

Patients’ demographic, clinical, and hospital-related characteristics are summarized in Table 3. Among the included patients, totally 2303 **(**11.2%) were frail and 18,224 (88.8%) were non-frail. The majority were aged 46–64 years (51.6%), female (68.0%), White (60.1%), with income at the lowest quartile (28.3%). The mean (SE, min-max) NIS-SAH severity score was 4.6 (0.1, 0–19). Further, there were higher proportion of patients older than 65 years in frailty group than non-frailty group (33.8% vs 23.0%, *p* < 0.001). The distribution of all characteristics was significantly different between the frailty and non-frailty groups (all *P* ≤ 0.006), except for sex, aneurysm treatment, vasospasm, hospital bedsize and hospital location.

**Table 3:** Characteristic of hospitalized patients with aSAH

Characteristics	Total	Frailty	Non-frailty	***P***-value
**Demographics**				
Age, mean ± SE	54.8 ± 0.1	58.0 ± 0.3	54.4 ± 0.1	**< 0.001**
18–45	5001 (24.4)	394 (17.2)	4607 (25.3)	**< 0.001**
46–64	10,597 (51.6)	1176 (51.0)	9421 (51.7)	**< 0.001**
≥ 65	4929 (24.0)	733 (31.8)	4196 (23.0)	**< 0.001**
Female, sex	13,956 (68.0)	1538 (66.8)	12,418 (68.2)	0.175
Race				**< 0.001**
White	10,113 (60.1)	1183 (62.8)	8930 (59.7)	
Black	2778 (16.5)	324 (17.1)	2454 (16.5)	
Hispanic	2281 (13.5)	205 (10.8)	2076 (13.8)	
Asian or Pacific Islander	778 (4.6)	65 (3.4)	713 (4.8)	
Other	890 (5.3)	111 (5.9)	779 (5.2)	
Household income				**0.004**
Quartile 1	5658 (28.3)	678 (30.2)	4980 (28.0)	
Quartile 2	5235 (26.2)	619 (27.7)	4616 (26.0)	
Quartile 3	4838 (24.3)	532 (24.0)	4306 (24.3)	
Quartile 4	4235 (21.2)	401 (18.0)	3834 (21.6)	
aSAH severity, mean ± SE (min-max)	4.6 ± 0.1 (0–19.0)	6.9 ± 0.1 (0–17.7)	4.4 ± 0.1 (0–19.0)	**< 0.001**
Aneurysm Treatment				0.058
Coiling	12,223 (59.6)	1422 (61.7)	10,801 (59.4)	
Microsurgical clipping	8304 (40.4)	881 (38.3)	7423 (40.6)	
**Disease/Comorbidities**				
Cerebral herniation	1166 (5.8)	182 (7.9)	984 (5.5)	**< 0.001**
Cerebral edema	3050 (14.9)	485 (21.1)	2565 (14.2)	**< 0.001**
Diabetes	2304 (11.2)	325 (14.0)	1979 (10.8)	**< 0.001**
Hypertension	12,579 (61.3)	1518 (65.8)	11,061 (60.7)	**0.001**
Peripheral vascular disorders	1384 (6.7)	196 (8.5)	1188 (6.5)	**< 0.001**
Vasospasm	384 (1.9)	47 (2.0)	337 (1.8)	0.502
**Provider**				
Hospital Bedsize				0.088
Small	756 (3.5)	79 (3.3)	677 (3.6)	
Medium	2550 (12.6)	241 (10.6)	2309 (12.8)	
Large	17,031 (83.9)	1956 (86.1)	15,075 (83.6)	
Hospital Location				0.567
Rural	199 (0.9)	17 (0.7)	182 (1.0)	
Urban	20,138 (99.1)	2259 (99.3)	17,879 (99.0)	
Hospital teaching status				**0.006**
Non-teaching	2459 (11.8)	218 (9.3)	2241 (12.1)	
Teaching	17,878 (88.2)	2058 (90.7)	15,820 (87.9)	

Categorical data were presented as unweighted counts (weighted percentage)

*aSAH* aneurysmal subarachnoid hemorrhage

### Effect of frailty on the outcome

The result of univariate and multivariate regression analysis for the association between frailty and outcomes are shown in Tables 4, 5, 6 and 7. In univariate analysis, patients with frailty was significantly associated with decreased risk of in-hospital mortality rate (OR: 0.76, 95% CI: 0.64–0.89). In addition, frailty was significantly associated with increased risk of discharge to institutional care (OR: 4.00, 95% CI: 3.46–4.62), tracheostomy or gastrostomy tube placement (OR: 5.56, 95% CI: 4.88–6.33), and occurrence of any postoperative complication (OR: 5.76, 95% CI: 4.62–7.18) as compared to patients without frailty.

**Table 4:** Associations between frailty, other variables and in-hospital mortality

Variables	In-hospital mortality			
	OR (95% CI)	***p***-value	aOR (95% CI)	***p***-value
**Frailty (vs No)**	**0.76 (0.64, 0.89)**	**< 0.001**	**0.40 (0.33, 0.49)**	**< 0.001**
**Demographics**				
Age (vs 18–45)				
46–64	**1.49 (1.33, 1.68)**	**< 0.001**	**1.27 (1.10, 1.46)**	**0.001**
≥ 65	**2.81 (2.48, 3.18)**	**< 0.001**	**2.24 (1.93, 2.61)**	**< 0.001**
Gender (vs Female)				
Male	0.98 (0.90, 1.07)	0.625		
Race (vs White)				
Black	**0.63 (0.55, 0.73)**	**< 0.001**	**0.70 (0.59, 0.82)**	**< 0.001**
Hispanic	**0.73 (0.63, 0.85)**	**< 0.001**	**0.74 (0.63, 0.87)**	**< 0.001**
Asian or Pacific Islander	0.99 (0.80, 1.23)	0.958	0.79 (0.62, 1.01)	0.063
Other	0.84 (0.68, 1.05)	0.123	0.82 (0.64, 1.05)	0.113
Income (vs Quartile 1)				
Quartile 2	0.94 (0.85, 1.05)	0.291		
Quartile 3	1.01 (0.90, 1.13)	0.868		
Quartile 4	0.93 (0.82, 1.05)	0.236		
aSAH severity	**1.23 (1.22, 1.25)**	**< 0.001**	**1.23 (1.22, 1.25)**	**< 0.001**
Treatment (vs Coiling)				
Microsurgical clipping	**0.87 (0.80, 0.96)**	**0.004**	0.98 (0.87, 1.09)	0.685
**Disease/Comorbidities (vs No)**				
Cerebral herniation	**4.18 (3.48, 5.02)**	**< 0.001**	**2.28 (1.84, 2.82)**	**< 0.001**
Cerebral edema	**2.36 (2.11, 2.64)**	**< 0.001**	**1.32 (1.14, 1.53)**	**< 0.001**
Diabetes	**1.21 (1.08, 1.36)**	**0.002**	1.03 (0.89, 1.19)	0.728
Hypertension	1.05 (0.97, 1.14)	0.255		
Peripheral vascular disorders	1.14 (0.98, 1.33)	0.083		
Vasospasm	**1.43 (1.10, 1.86)**	**0.007**	**1.39 (1.02, 1.90)**	**0.036**
**Provider**				
Bedsize (vs Small)				
Medium	1.24 (0.93, 1.66)	0.142		
Large	1.11 (0.85, 1.45)	0.453		
Location (vs Rural)				
Urban	**0.88 (0.79, 0.99)**	**0.038**	**1.27 (1.05, 1.54)**	**0.013**
Teaching status (vs Non)				
Teaching	**0.67 (0.60, 0.74)**	**< 0.001**	**0.64 (0.56, 0.74)**	**< 0.001**

**Table 5:** Associations between frailty, other variables and discharge to institutional care

Variables	Discharge to institutional care			
	OR (95% CI)	***p***-value	aOR (95% CI)	***p***-value
**Frailty (vs No)**	**4.00 (3.46, 4.62)**	**< 0.001**	**2.50 (2.10, 2.97)**	**< 0.001**
**Demographics**				
Age (vs 18–45)				
46–64	**1.86 (1.72, 2.02)**	**< 0.001**	**1.64 (1.50, 1.80)**	**< 0.001**
≥ 65	**5.56 (5.05, 6.11)**	**< 0.001**	**5.14 (4.57, 5.79)**	**< 0.001**
Gender (vs Female)				
Male	**0.88 (0.82, 0.93)**	**< 0.001**	0.96 (0.89, 1.05)	0.377
Race (vs White)				
Black	**0.87 (0.79, 0.96)**	**0.006**	1.03 (0.92, 1.15)	0.603
Hispanic	**0.63 (0.57, 0.71)**	**< 0.001**	**0.64 (0.56, 0.73)**	**< 0.001**
Asian or Pacific Islander	**0.84 (0.70, 1.00)**	**0.049**	**0.78 (0.63, 0.96)**	**0.017**
Other	**0.82 (0.71, 0.95)**	**0.010**	**0.82 (0.68, 1.00)**	**0.048**
Income (vs Quartile 1)				
Quartile 2	1.04 (0.95, 1.13)	0.381		
Quartile 3	0.98 (0.89, 1.06)	0.568		
Quartile 4	0.98 (0.87, 1.10)	0.707		
aSAH severity	**1.21 (1.20, 1.22)**	**< 0.001**	**1.19 (1.18, 1.21)**	**< 0.001**
Treatment (vs Coiling)				
Microsurgical clipping	**1.10 (1.02, 1.18)**	**0.012**	**1.29 (1.17, 1.42)**	**< 0.001**
**Disease/Comorbidities (vs No)**				
Cerebral herniation	**2.37 (1.86, 3.01)**	**< 0.001**	1.22 (0.93, 1.60)	0.143
Cerebral edema	**1.91 (1.70, 2.15)**	**< 0.001**	**1.41 (1.22, 1.62)**	**< 0.001**
Diabetes	**1.71 (1.56, 1.88)**	**< 0.001**	**1.38 (1.22, 1.56)**	**< 0.001**
Hypertension	**1.33 (1.25, 1.42)**	**< 0.001**	1.07 (0.99, 1.16)	0.109
Peripheral vascular disorders	**1.34 (1.19, 1.50)**	**< 0.001**	**1.16 (1.00, 1.34)**	**0.047**
Vasospasm	1.19 (0.95, 1.50)	0.126		
**Provider**				
Bedsize (vs Small)				
Medium	1.08 (0.84, 1.40)	0.535		
Large	1.03 (0.81, 1.31)	0.817		
Location (vs Rural)				
Urban	0.99 (0.89, 1.12)	0.924		
Teaching status (vs Non)				
Teaching	**0.87 (0.79, 0.96)**	**0.007**	**0.83 (0.72, 0.94)**	**0.005**

**Table 6:** Associations between frailty, other variables and tracheostomy or gastrostomy tube placement

Variables	Tracheostomy or gastrostomy tube placement			
	OR (95% CI)	***p***-value	aOR (95% CI)	***p***-value
**Frailty (vs No)**	**5.56 (4.88, 6.33)**	**< 0.001**	**4.41 (3.81, 5.10)**	**< 0.001**
**Demographics**				
Age (vs 18–45)				
46–64	**1.50 (1.35, 1.66)**	**< 0.001**	**1.33 (1.17, 1.52)**	**< 0.001**
≥ 65	**2.55 (2.28, 2.85)**	**< 0.001**	**2.08 (1.79, 2.41)**	**< 0.001**
Gender (vs Female)				
Male	0.99 (0.92, 1.07)	0.845		
Race (vs White)				
Black	**1.25 (1.11, 1.39)**	**< 0.001**	**1.46 (1.28, 1.67)**	**< 0.001**
Hispanic	0.96 (0.85, 1.09)	0.557	1.06 (0.91, 1.24)	0.458
Asian or Pacific Islander	1.09 (0.91, 1.32)	0.355	1.10 (0.89, 1.37)	0.379
Other	1.11 (0.92, 1.35)	0.273	1.06 (0.84, 1.34)	0.627
Income (vs Quartile 1)				
Quartile 2	0.94 (0.84, 1.04)	0.217	0.92 (0.81, 1.04)	0.193
Quartile 3	**0.85 (0.76, 0.95)**	**0.004**	**0.85 (0.74, 0.98)**	**0.029**
Quartile 4	**0.87 (0.77, 0.98)**	**0.021**	0.91 (0.79, 1.05)	0.209
aSAH severity	**1.22 (1.21, 1.24)**	**< 0.001**	**1.22 (1.20, 1.23)**	**< 0.001**
Treatment (vs Coiling)				
Microsurgical clipping	**1.11 (1.02, 1.21)**	**0.013**	**1.31 (1.18, 1.45)**	**< 0.001**
**Disease/Comorbidities (vs No)**				
Cerebral herniation	**2.01 (1.73, 2.34)**	**< 0.001**	0.92 (0.75, 1.13)	0.453
Cerebral edema	**1.85 (1.67, 2.05)**	**< 0.001**	**1.18 (1.04, 1.34)**	**0.012**
Diabetes	**1.63 (1.47, 1.80)**	**< 0.001**	**1.31 (1.14, 1.49)**	**< 0.001**
Hypertension	1.06 (0.97, 1.16)	0.198		
Peripheral vascular disorders	**1.29 (1.13, 1.48)**	**< 0.001**	**1.17 (0.98, 1.40)**	**0.074**
Vasospasm	0.97 (0.75, 1.26)	0.815		
**Provider**				
Bedsize (vs Small)				
Medium	**1.37 (1.02, 1.83)**	**0.034**		
Large	**1.36 (1.04, 1.78)**	**0.022**		
Location (vs Rural)				
Urban	**1.29 (1.04, 1.61)**	**0.022**	0.95 (0.87, 1.05)	0.309
Teaching status (vs Non)				
Teaching	0.96 (0.85, 1.08)	0.495

**Table 7:** Associations between frailty, other variables and postoperative complications

Variables	Postoperative complications			
	OR (95% CI)	***p***-value	aOR (95% CI)	***p***-value
**Frailty (vs No)**	**5.76 (4.62, 7.18)**	**< 0.001**	**3.29 (2.55, 4.25)**	**< 0.001**
**Demographics**				
Age (vs 18–45)				
46–64	**1.49 (1.38, 1.60)**	**< 0.001**	**1.18 (1.08, 1.29)**	**< 0.001**
≥ 65	**2.82 (2.54, 3.14)**	**< 0.001**	**1.79 (1.57, 2.05)**	**< 0.001**
Gender (vs Female)				
Male	0.98 (0.91, 1.06)	0.625		
Race (vs White)				
Black	**0.82 (0.74, 0.92)**	**< 0.001**	**0.88 (0.77, 0.99)**	**0.039**
Hispanic	0.87 (0.75, 1.01)	0.063	0.96 (0.83, 1.12)	0.640
Asian or Pacific Islander	**1.26 (1.01, 1.57)**	**0.039**	1.24 (0.97, 1.59)	0.083
Other	0.92 (0.78, 1.09)	0.334	0.92 (0.77, 1.09)	0.328
Income (vs Quartile 1)				
Quartile 2	1.07 (0.97, 1.18)	0.183		
Quartile 3	1.00 (0.91, 1.10)	0.961		
Quartile 4	**1.14 (1.01, 1.28)**	**0.028**		
aSAH severity	**1.30 (1.28, 1.32)**	**< 0.001**	**1.27 (1.25, 1.30)**	**< 0.001**
Treatment (vs Coiling)				
Microsurgical clipping	**0.83 (0.76, 0.91)**	**< 0.001**	**0.89 (0.80, 0.98)**	**0.015**
**Disease/Comorbidities (vs No)**				
Cerebral herniation	**3.51 (2.69, 4.56)**	**< 0.001**	1.29 (0.97, 1.72)	0.076
Cerebral edema	**3.12 (2.66, 3.65)**	**< 0.001**	**1.93 (1.63, 2.28)**	**< 0.001**
Diabetes	**1.65 (1.45, 1.86)**	**< 0.001**	**1.24 (1.06, 1.44)**	**0.006**
Hypertension	**1.53 (1.42, 1.66)**	**< 0.001**	**1.36 (1.24, 1.49)**	**< 0.001**
Peripheral vascular disorders	**2.04 (1.73, 2.41)**	**< 0.001**	**2.08 (1.64, 2.63)**	**< 0.001**
Vasospasm	**1.77 (1.30, 2.40)**	**< 0.001**	0.91 (0.58, 1.41)	0.663
**Provider**				
Bedsize (vs Small)				
Medium	**0.60 (0.44, 0.81)**	**< 0.001**	**0.55 (0.43, 0.71)**	**< 0.001**
Large	**0.72 (0.54, 0.95)**	**0.02**	**0.69 (0.55, 0.87)**	**0.001**
Location (vs Rural)				
Urban	0.95 (0.83, 1.08)	0.396		
Teaching status (vs Non)				
Teaching	**0.86 (0.75, 0.98)**	**0.027**	**0.79 (0.68, 0.91)**	**0.001**

The significant variables present in univariate analysis were included in the multivariate analysis, results of the multivariate analysis revealed that frailty remained significantly associated with a lower risk of dying in hospital (aOR: 0.40, 95% CI: 0.33–0.49) and higher risk of discharge to institutional care (aOR: 2.50, 95% CI: 2.10–2.97), tracheostomy or gastrostomy tube replacement (aOR: 4.41, 95% CI: 3.81–5.10) and postoperative complications (aOR: 3.29, 95% CI: 2.55–4.25) compared with the non-frailty patients.

### Effect of frailty on outcomes by age and aneurysm treatment

Table 8 summarizes the results of further examination of associations between frailty and outcomes stratified by different age groups and types of aneurysm repair. Among younger patients (< 65 years), frailty patients were significantly associated with increased risk of discharge to institutional care (aOR: 2.74, 95% CI: 2.22–3.39), tracheostomy or gastrostomy tube placement (aOR: 5.08, 95% CI: 4.29–6.00), postoperative complications (aOR: 3.20, 95% CI: 2.44–4.21) and had significantly lower odds of in-hospital mortality rate (aOR: 0.40, 95% CI: 0.32–0.51) as compared to the non-frailty group. Among older patients (≥ 65 years old), frail patients also had significantly higher odds of discharge to institutional care (aOR: 1.94, 5% CI: 1.49–2.52), tracheostomy or gastrostomy tube placement (aOR: 3.34, 95% CI: 2.70–4.14), postoperative complication (aOR: 3.71, 95% CI: 2.25–6.13) and had significantly lower odds of in-hospital mortality (aOR: 0.41, 95% CI: 0.31–0.54).

**Table 8:** Stratified analysis of associations between frailty on outcomes by age and aneurysm treatment

Variables	In-hospital mortality	Discharge to institutional care	Tracheostomy or gastrostomy tube placement	Postoperative complications
	aOR (95% CI)	aOR (95% CI)	aOR (95% CI)	aOR (95% CI)
***Age***				
Age < 65	**0.40 (0.32, 0.51)**	**2.74 (2.22, 3.39)**	**5.08 (4.29, 6.00)**	**3.20 (2.44, 4.21)**
Age ≥ 65	**0.41 (0.31, 0.54)**	**1.94 (1.49, 2.52)**	**3.34 (2.70, 4.14)**	**3.71 (2.25, 6.13)**
***Aneurysm Treatment***				
Coiling	**0.44 (0.35, 0.55)**	**2.54 (2.12, 3.06)**	**4.41 (3.74, 5.19)**	**3.24 (2.37, 4.43)**
Microsurgical Clipping	**0.34 (0.25, 0.46)**	**2.45 (1.89, 3.18)**	**4.46 (3.61, 5.50)**	**3.36 (2.38, 4.74)**

Adjusted for the covariates as same as the multivariate model in Tables 4, 5, 6 and 7 except the stratified variable

Among the subgroups of patients who received coiling embolization procedure during hospitalization, frailty patients were significantly associated with higher odds of discharge to institutional care (aOR: 2.54, 95% CI: 2.12–3.06), tracheostomy or gastrostomy tube placement (aOR: 4.41, 95% CI: 3.74–5.19), postoperative complication (aOR: 3.24, 95% CI: 2.37–4.43) and lower odds of dying in the hospital (aOR: 0.44, 95% CI: 0.35–0.55) as compared to non-frailty. Among those who received clipping, frailty patients also had significantly higher odds of discharge to institutional care (aOR: 2.45, 95% CI: 1.89–3.18), tracheostomy or gastrostomy tube placement (aOR: 4.46, 95% CI: 3.61–5.50) and postoperative complication (aOR: 3.36, 95% CI: 2.38–4.74) and lower odds of dying in hospital (aOR: 0.34, 95% CI: 0.25–0.46).

## Discussion

In this population-based study, a comparison of clinical characteristics and in-hospital outcomes of 15,560 hospitalized aSAH patients with and without frailty revealed that frailty significantly increases the risk of discharge to institutional care, tracheostomy, or gastrostomy tube placement, and postoperative complications after aneurysm repair surgery. Conversely, frailty significantly reduces the risk of in-hospital mortality. The effect of frailty on these four outcomes was even more significant among patients younger than 65 years than among elder aged 65 years and older. In addition, the effect of frailty on these four outcomes was slightly more significant among patients who received Clipping surgery to treat an aneurysm than among patients who received coiling embolization procedures.

This study’s results assistant with previous research showing that frailty significantly increases the risk of discharge to institutional care. Seamon et al. [26] investigated the effects of frailty on discharge location after acute stroke, showed that pre-stroke frailty was an indicator of inpatient rehabilitation discharge (i.e., to a rehabilitation or nursing care facility with 24-hour care) after controlling for age, stroke severity, and comorbidities. Mclntyre et al. [27] found that frail patients with angiogram-negative subarachnoid hemorrhage were 6.2 times less likely to be discharged home. We believe that the possible reason is that patients with frailty have a slower recovery rate, and they need institutional care after the operation and correct the factors that contribute to the development of frailty.

Postoperative complications, at least one, were noted in about 69% of frail patients in the present study, compared to 48% in non-frail patients. Critically ill patients with frailty more often required organ support like mechanical ventilation, vasopressors, dialysis, and transfusion than non-frail patients [13]. It is in line with the findings of our present study that frail patients will significantly increase the risk of tracheostomy or gastrostomy tube placement. A strong association was shown between frailty and oncologic neurosurgery complications such as coma, stroke, and neurologic deficit, and there is a difference in the degree of frailty [10]. Those authors explained these results as a reflection of higher preoperative partial functional dependence and hemiplegia rates in the low frailty group. Diminished cognition has also been shown to be a post-stroke or post-aSAH complication. Pre-stroke frailty was suggested to be a moderator of post-stroke cognition [12], and survivors of aSAH commonly reported to experience long-term cognitive deficits [28]. The lack of pertinent data in the NIS database did not evaluate the effect of frailty on cognitive function. However, this study showed that patients classified as frailty meet the criteria of malnutrition. The surgical intervention itself may trigger a sequence of inflammatory reactions, and the body will need a certain amount of energy to respond. Therefore, when the patient is malnourished, the body cannot respond appropriately, leading to postoperative complications.

We unexpectedly found that frailty significantly reduces the risk of in-hospital mortality. The results is similar as in the previous study that also focused on the effects of frailty in aSAH [29]. They reported that frailty was not an independent predictor of mortality and whereas age has a greater impact. It may be because medical staff expects surgery to bring more adverse outcomes to frail patients to pay more attention to them. The dataset does not provide data of deaths after discharge. In the present analysis, frailty did double the risk of discharge to long-term facilities. It is possible that some deaths occurred after discharge in a short time at such facilities. Nevertheless, further research is needed to clarify this issue.

The present study also showed that frailty has a more significant effect on young adults (<65y) than older adults (≥65y). The effect of frailty on different surgical methods is not much different. Patients diagnosed with aSAH have traditionally been aged between 40 and 60 [30]. Advanced chronological age also has been regarded as an important prognostic indicator of poor outcomes after aSAH [20, 31]. However, in the present study, although increasing age is correlated with patients’ outcomes, frailty was an independent predictor of outcomes and must still be considered for decision-making and perioperative care planning. In addition, recent studies, including our present study, have shown exciting results for younger adult patients. While frailty is associated with advanced age [1, 4], and causally influenced by age, institutionalization, and death [32], younger critically ill patients were shown to be at greater risk of mortality and higher re-hospitalization rates at 1 year after discharge than those who survived critical illness [33]. A recent study suggests that frailty is common among younger critically ill patients and younger adults admitted to emergency general surgical units, potentially leading to adverse short and long-term outcomes [33, 34]. Results of these studies agree with our finding that the potential effect of frailty on unfavorable outcomes was more significant among younger adults than elders, which emphasizes the need to identify and characterize aSAH in younger adult patients. We found that patients who are classified as frailty are because they meet the criteria of malnutrition. Due to the reduced metabolic needs of the elderly, the impact of malnutrition may be less than patients in the prime of life. Further research is needed to clarify it.

For many years, the lack of an absolute definition of frailty and a standard and valid screening method contributed to the absence of supportive interventions to ameliorate the effects of frailty on outcomes of surgeries and critical illness in older adults [1, 18]. Until recently, measures of frailty such as mobility, nutritional status, and disability were not typically included in surgical risk scores [35], regardless of the acknowledged impact of frailty on surgical outcomes [6, 8]. Several instruments are readily available for physiological and surgical risk assessment, including patient-related factors that help considerably predict operative morbidity and mortality. The Fried frailty phenotype criteria seem worthwhile based on unintentional weight loss, exhaustion, decreased grip strength, decreased walking speed, and low physical activity—all easily measured preoperatively [1]. The Johns Hopkins Adjusted Clinical Groups (ACG) frailty-defining diagnoses indicator was developed and validated fairly recently and assessed frailty according to ICD-9 codes assigned at admission [22]. The ACG has been increasingly applied to explore the association between frailty and predict postoperative outcomes in various surgical settings based on data from administrative databases [11, 23, 36, 37]. The ACG index was used effectively in the present study, although it does not distinguish between degrees of frailty. Overall, the usefulness and reliability of risk assessment indices appear to depend on the given patient population, the surgical procedure or critical condition being treated, and the availability of pertinent clinical data.

### Strengths and limitations

This study is the first report to explore an association between frailty as measured by the ACG frailty indicator and the outcome after aSAH treatment. The primary strength of this study is the comprehensive NIS database, which mirrors the US population and allows results to be generalized nationwide. The severity of aSAH was graded by a standardized scale. Other critical confounding variables such as patients’ comorbidities and hospital characteristics were considered and adjusted for analysis. Several limitations are still noted, including the retrospective analysis of data, which cannot exclude biases and limits inferences of causation. The Johns Hopkins ACG frailty-defining index used in this study relies on ICD-9 codes. Frailty may therefore be underestimated due to the under-coding of frailty-defining diagnoses, and the ACG index is suitable only for use with an administrative database, but it does not distinguish degrees of frailty. Although the ICD-9 diagnosis coding system was used to identify different categories of comorbidities in the included patients, the severity of individual comorbidities was not available. The NIS database also does not provide patients’ follow-up data after discharge, precludes evaluating late or long-term morbidity and mortality. The lack of other confounding variables not collected by the NIS like the location of ruptured aneurysms, operation time, laboratory parameters, and medical treatments may complicate the analysis and limit the interpretation of results. A further prospective cohort study of patients with aSAH stratified by age and degree of frailty is necessary to confirm the present study results.

## Conclusion

Frailty is significantly related to the increased risk of discharge to institutional care, tracheostomy or gastrostomy tube placement, and postoperative complications but the reduced in-hospital mortality rate after aneurysm repair. In addition, the relation of frailty on outcomes appears to be greater among younger adults than older adults. Thus, baseline frailty evaluation in aSAH patients provides valuable information for preoperative risk stratification and may lead to more effective care planning for this patient population.

## Data Availability

The datasets used during the current study are available in this article.

## Abbreviations

NIS: National Inpatient Sample
AHA: American Hospital Association
HCUP: Healthcare Cost and Utilization Project
NIH: National Institutes of Health
AVM: Cerebral arteriovenous malformation
TIA: Transient cerebral ischemia
aSAH: Aneurysmal subarachnoid hemorrhage
AHRQ: Healthcare Research and Quality
